# Maternal–Fetal Complications in Renal Colic during Pregnancy: A Scoping Review

**DOI:** 10.3390/jcm13185515

**Published:** 2024-09-18

**Authors:** Paulina Machura, Jakub S. Gąsior, Michał Ciebiera, Sylwia Dąbkowska, Diana Massalska

**Affiliations:** 1 Second Department of Obstetrics and Gynecology, Center of Postgraduate Medical Education, ul. Inflancka 6, 00-189 Warsaw, Poland; 2 Warsaw Institute of Women’s Health, ul. Inflancka 6, 00-189 Warsaw, Poland; 3 Department of Pediatric Cardiology and General Pediatrics, Medical University of Warsaw, 02-091 Warsaw, Poland

**Keywords:** pregnancy, renal, complications

## Abstract

Renal colic is one of the most common non-obstetric causes of hospitalization in pregnant women. Its management is often a challenge for obstetricians/gynecologists, urologists and neonatologists due to the complexity of the problem. The aim of this study was to analyze the possible maternal–fetal complications in renal colic during pregnancy. The authors performed a scoping review of the current literature regarding the analyzed issues. The review was conducted using the PubMed/MEDLINE and Web of Science databases. The search generated a total of 237 articles, out of which 7 original studies were ultimately included in the scoping review. In the women affected by renal colic, the incidence of perinatal complications such as urinary tract infections (UTIs), premature rupture of membranes (pPROM), and preterm birth is markedly higher than reported in the general population of pregnant women. Data regarding the recurrence of other perinatal complications such as gestational diabetes mellitus (GDM), pregnancy-induced hypertension (PIH), preeclampsia (PE), and intrauterine growth restriction (IUGR) are scarce and ambiguous. Further research on these issues is needed to improve the perinatal outcomes of the affected pregnancies.

## 1. Introduction

Renal colic is one of the most common non-obstetric causes of hospitalization during pregnancy associated with an increased risk of maternal–fetal complications [1,2,3,4,5,6,7]. The incidence of renal colic was estimated at around 1 in 1500 pregnant women, while complicated renal colic was found to affect 1 in 3300 pregnancies. The first symptoms usually occur in the second or third trimester [8]. Renal colic is defined as abdominal pain caused by hydronephrosis, which may be physiological during pregnancy, or caused by kidney stones. Hydronephrosis is a consequence of adaptive changes in the urinary system during pregnancy. An elevated progesterone level has a relaxing effect on the smooth muscles of the ureters, leading to their hypotonia and decreased peristalsis, which contributes to the dilatation of the upper urinary tract and hydronephrosis. Hydronephrosis most commonly occurs on the right side, which is related to the rotation of the enlarged uterus compressing the ureter and the pelvic venous vessels [1]. The occurrence of asymptomatic hydronephrosis is reported in over 90% of pregnant women. Exceptionally (in around 0.2–3% of cases), it generates colic [9]. Hydronephrosis caused by urolithiasis affects approximately 0.05% of pregnancies. The diagnostic options for renal stones, especially imaging methods, are severely limited in pregnancy, which may delay diagnosis and generate a higher risk of maternal–fetal complications [10,11].

Therefore, effective, multidisciplinary care in such cases is crucial for preserving maternal–fetal well-being [9,12,13]. Conservative treatment, including adequate intravenous hydration, antispasmodic and analgesic drugs, and, if a urinary tract infection is suspected, appropriate antibiotic therapy, is sufficient in around 70 to 80% of cases [14,15]. In complicated renal colic, defined as progressive hydronephrosis, septic infection, renal failure, bilateral inflammatory uropathy, or a single kidney obstruction, surgical procedures such as DJ stent implantation, ureteroscopy, or percutaneous nephrostomy (PCN) may be necessary [3,16]. The objective of this scoping review was to estimate the risk of maternal–fetal complications in renal colic during pregnancy.

## 2. Materials and Methods

The scientific literature was screened for articles analyzing renal colic in pregnancy according to the updated guidelines for scoping reviews—the Preferred Reporting Items for Systematic Reviews and Meta-Analyses (PRISMA) 2020 statement [17,18]—with an extension designed for scoping reviews [19] using the PubMed, Web of Science, and Cumulative Index to Nursing and Allied Health Literature (CINAHL) databases. The databases were explored using the mesh terms “renal colic” and “pregnancy” (“renal colic” [Title/Abstract] AND “pregnancy” [Title/Abstract]), and the search included the following limitations: (i) studies limited to humans; (ii) publications in English; (iii) empirical investigations, i.e., studies involving active data collection; and (iv) publication date from 1990 to 2024. The following types of articles were excluded: conference abstracts, non-English articles, articles with no available abstract, meta-analyses, expert opinions, reviews, single case reports, and articles analyzing the general population, not pregnant women or women with renal colic during pregnancy. Two researchers performed identification and screening procedures independently (PM/JSG). An initial identification of the results of the search was performed on the titles and abstracts, with an analysis of the full texts being performed in cases that were uncertain. In an effort to expand the search, the references of the selected articles were examined for studies not found in the database. Two researchers independently assessed the selected studies for eligibility (PM/JSG). In case of differences of opinions, the matters were discussed with other co-authors and resolved in this way.

Through the use of the PICO approach (patient population or disease being addressed (P), interventions or exposure (I), comparator group (C), outcome or endpoint (O) [17]), the following information was extracted from each study:

(1) First author and year of publication;
(2) Participant characteristics;
(3) Maternal and fetal complications, including the following:
  I. Pregnancy-induced hypertension (PIH);
  II. Preeclampsia (PE);
  III. Gestational diabetes mellitus (GDM);
  IV. Premature rupture of membranes (pPROM);
  V. Urinary tract infection (UTI);
  VI. Urosepsis;
  VII. Cesarean section delivery;
  VIII. Premature birth;
  IX. Intrauterine fetal demise;
  X. NICU (neonatal intensive care unit) admission;
  XI. Intrauterine growth restriction (IUGR).

## 3. Results

### 3.1. Selection of the Studies

**Figure 1:**
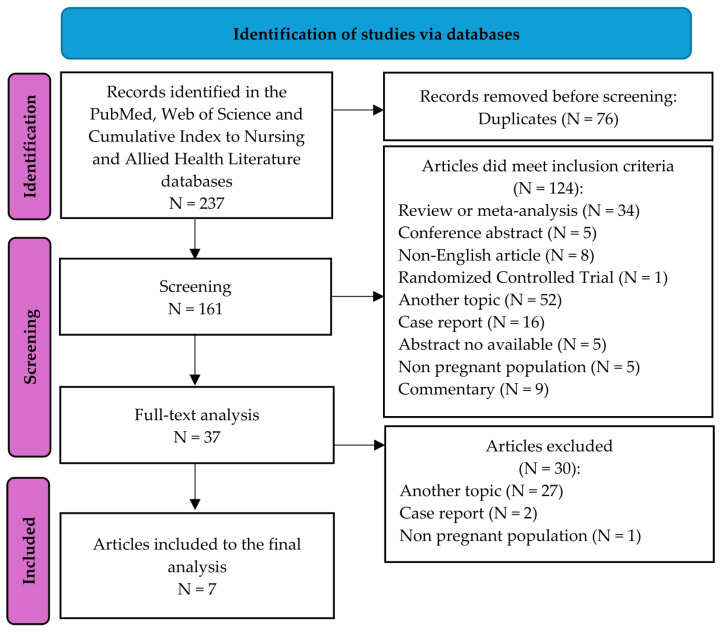
PRISMA 2020 flow diagram [17].

The search generated a total of 237 articles from the databases (Figure 1), of which 76 were removed due to duplication. Of the remaining 161 articles, 124 did not meet the inclusion criteria, leaving 37 to undergo a full-text analysis. Finally, 31 more articles were discarded for specified reasons (see Figure 1), leaving a total of 7 studies to be included in the review [1,2,3,4,5,6,7] (Table S1). No additional studies were identified by checking the references within these seven studies.

### 3.2. Characteristics of the Study Group

The analyzed group included 12,086 women diagnosed with renal colic during pregnancy. The mean maternal age varied between 23 and 32 years between studies [1,2,3,5,6,7]. The mean gestational age of analyzed cases ranged between 22 and 39 weeks in the individual included publications [2,4,7].

### 3.3. Maternal Complications

The most frequent maternal complications in pregnancies affected by renal colic included urinary tract infections (UTIs), reported in 5.46–23.7% of women [1,2,4,6] and leading to maternal sepsis in 0–0.85% of cases [3,5].

pPROM seemed to occur more frequently in the women with renal colic, but its occurrence ranged broadly between 0.63 and 15.38% in individual studies [1,4,6].

The reported frequency of cesarean deliveries in women affected by renal colic was between 30.0 and 38% [1,2,6,7]. Data regarding the occurrence of other perinatal complications such as PIH, PE and GDM were scarce and ambiguous (summarized in Table 1). PIH was reported in 3.85–22% of pregnancies, PE in 5.77–16%, and GDM in 3.66–18% [1,6,7]. The frequency was comparable to that in the general population. However, one study showed that the incidence of those metabolic outcomes was markedly higher in the group of patients with urolithiasis (frequencies summarized in Table 1).

### 3.4. Fetal (Neonatal) Complications

Premature delivery was the most frequent fetal (neonatal) complication of renal colic during pregnancy. The frequency of premature births in the analyzed studies ranged between 2.44 and 30.77% of pregnancies [1,2,3,4,6,7]. The frequency of hospitalizations in neonatal intensive care units (NICUs) was assessed in two studies, and the range was 10.0–19.23% [1,7]. The occurrence of intrauterine fetal demise ranged between 0 and 0.37% of pregnancies [1,6]. IUGR occurred in 1.97 to 9.62% of fetuses [1,6,7].

## 4. Discussion

Renal colic is the most frequently reported symptom in pregnant women with urolithiasis. A retrospective analysis by Chan et al. showed that flank pain was reported by 89% of pregnant women with urolithiasis. The same study showed that non-visible hematuria and visible hematuria occurred in 95% and 20% of patients, respectively. In addition, other nonspecific symptoms reported by patients included nausea, vomiting, and dysuria [13]. Renal colic in pregnancy is a relatively rare but possibly serious condition. A review of the literature revealed that there is a scarcity of data regarding obstetric and fetal (neonatal) complications in pregnancies complicated by renal colic. In addition, the majority of original papers, as well as those that were included in our analysis, referred to pregnant women who underwent specific urological procedures. Understanding the risk of perinatal complications in this group of patients is crucial for urologists and obstetricians to improve the quality of pregnancy management.

The available literature revealed an increased risk of perinatal complications related to renal colic in pregnancy. Some analyses showed that urolithiasis and secondary renal colic during pregnancy were associated with an up to 30% increase in the risk of preterm delivery. It was also observed that kidney stones during pregnancy were significantly positively associated with pPROM, IUGR, cesarean delivery, UTI, PE, sepsis, and metabolic diseases such as PIH or GDM. Moreover, newborns of mothers with kidney stones were at a significantly greater risk of complications of prematurity, and they were more often hospitalized in NICUs [6,7,12,20,21,22,23,24,25].

Our literature review did not confirm some of those reports. Only seven studies met the inclusion criteria of this literature review. The study groups were heterogeneous in the context of the number of patients. Moreover, studies revealed differences in the maternal age and gestational age of the included cases [1,2,3,4,5,6], which makes the analyzed results hard to compare.

### 4.1. Premature Delivery and Preterm Rupture of Membranes (pPROM)

According to the performed literature review, premature birth was the most common neonatal complication in pregnancies affected by renal colic [1,3,6,7]. Also, pPROM was reported in those pregnancies at an increased frequency. A meta-analysis by Zhou et al. using the PubMed, Embase, and Cochrane databases up to 2020 showed that renal stones in pregnancy might increase the risk of preterm birth compared to the control group, but there was no association between urolithiasis in pregnant women and the risk of pPROM and increased neonatal mortality [26]. Interestingly, the authors of a systematic review and meta-analysis of 4.7 million pregnancies confirmed that renal colic was not significantly associated with pPROM or infant mortality [22]. In contrast, Lewis et al. estimated that pregnant women with renal colic had a significantly higher rate of pPROM [25].

Preterm birth is defined as birth before 37 weeks of pregnancy. The mechanism underlying the connection between urolithiasis and premature delivery is still uncertain, but it seems that several factors are involved. Some authors speculated that earlier deliveries due to the enhanced risk of experiencing obstetric complications, such as preeclampsia and placental abruption, might explain the higher frequency of preterm delivery [6]. Surgical procedures performed in pregnant patients with renal colic, such as ureteroscopy and ureteral stent placement, may stimulate the cervix and induce uterine contractions. Long operation time was found to be a particularly important factor that significantly affected the risk of premature uterine contractions [27]. Moreover, it was shown that the prostanoids produced during ureteral obstruction had a major role in the modulation of contractility and promoted labor by stimulating uterine contractions and ripening the cervix. In addition, vomiting and subsequent dehydration may contribute to premature birth by reducing blood flow through the uterus and the secretion of antidiuretic hormone, oxytocin, and prostaglandins. Urolithiasis during pregnancy may also increase the risks of infection, such as urinary tract infection, urosepsis; the infection itself may induce premature labor [20].

### 4.2. Urinary Tract Infections

Urinary tract infection (UTI) is the most common maternal complication according to the performed analysis. The definition of UTI is the presence of microbial pathogens in any part of the urinary tract, including the kidneys, ureters, bladder, or urethra. The available data suggested that an inflammation or infection of the vagina or urinary tract might be the key cause of up to 25% of cases of preterm birth [26]. In addition, bacteria from the genital tract can penetrate through intact mucous membranes and invade the amniotic and decidual cavities, which in turn may cause chorioamnionitis and an acute inflammatory reaction associated with premature rupture of membranes [28].

Moreover, in the group of pregnant women with UTI, other perinatal complications were observed much more often—IUGR, PE, cesarean deliveries [29], neonatal complications such as sepsis, pneumonia (specifically, group B Streptococcus [GBS] infection), stillbirth, and a higher rate of hospitalizations in the NICU [30].

The main causes of renal colic, i.e., kidney stones or the compression of the ureters by the pregnant uterus, may cause blockage of the ureter, leading to the development of hydronephrosis and proximal infection. Conversely, urinary tract infections may increase urine pH, potentially leading to stone formation [29].

The most frequently identified pathogens in the urine of pregnant women with UTIs included *Escherichia coli*, *Proteus mirabilis*, *Klebsiella pneumoniae*, *group B Streptococcus*, and *Staphylococcus saprophyticus*. Moreover, UTI in pregnancy may be due to atypical pathogens such as *Chlamydia trachomatis*, and *Ureaplasma urealyticum* [30]. Radu et al. analyzed the factors associated with an increased risk of urosepsis. They showed that hydronephrosis was one of the main factors in urosepsis during pregnancy due to gestational or reno-ureteral lithiasis. Moreover, the risk of urosepsis increased if a patient had second- or third-degree hydronephrosis [31].

To sum up, renal colic during pregnancy is associated with a higher risk of UTI, which may lead to pyelonephritis and urosepsis, which significantly increases the risk of perinatal complications for the mother and fetus.

### 4.3. Metabolic Complications

Tangren et al. assumed that stone formation was a marker of metabolic disease, and they found that GDM, PIH, and PE were more common in pregnant women with urolithiasis, which contributed to a higher risk of maternal complications. They also reported that first-trimester body mass index was significantly correlated with stone formation and hypertensive complications of pregnancy and systemic disorders, including diabetes and metabolic syndrome, in the general population [7]. Although the mechanism is unknown, it is supposed that urolithiasis may cause impaired renal function, which may contribute to an increased risk of hypertension or preeclampsia during pregnancy [6]. During pregnancy, numerous alterations occur in maternal metabolism and systemic hemodynamics. The functional changes include increased renal blood flow, mainly in the first and second trimester; an increased renal volume; and a glomerular filtration rate of approximately 50%. Physiological glycosuria, proteinuria, and alkaline urinary pH may occur [32,33]. The adaptive changes occurring during pregnancy are crucial for the proper course of pregnancy and, therefore, the well-being of the mother and fetus. Any renal dysfunction during pregnancy increases the risk of fetomaternal complications such as preeclampsia, premature delivery, and metabolic diseases. 

During pregnancy, numerous hormonal changes also occur, including increases in adrenocorticotropin (ACTH), vasopressin, cortisol, placental somatotropin, thyroid hormones, and natriuretic hormones such as aldosterone [34]. An increased glomerular filtration rate results in the production of urine containing higher concentrations of calcium and other prolithogenic factors such as uric acid and oxalate. Conversely, the secretion of substances inhibiting the formation of deposits, mainly citrate, magnesium, glycosaminoglycans, and acid glycoproteins, in the kidneys is reduced. In addition, pregnancy is associated with physiological hypercalciuria and an increased absorption of calcium in the gastrointestinal tract, mainly due to increased placental production of 1,25-dihydroxycholecalciferol [35,36]. Urinary calcium excretion may increase three-fold, which favors the formation of renal stones, which are mainly composed of calcium phosphates (74%), with the remainder being composed of calcium oxalates (26%) [35]. 

According to the literature, despite the numerous metabolic and hormonal changes which accompany pregnancy, the incidence of nephrolithiasis is not increased compared to that in non-pregnant women [25]. However, Thongprayoon et al. conducted a retrospective analysis and showed that pregnancy increased the risk of a first episode of symptomatic nephrolithiasis, most significantly at the time of delivery and persisting for approximately one year after delivery [36]. Kidney stones are also a risk factor for the development of diabetes. It is assumed that insulin resistance results in prolithogenic changes in urinary composition. Moreover, pregnant women with kidney stones were more commonly found to have insulin resistance before pregnancy, which was uncovered as a result of metabolic changes occurring physiologically during pregnancy [26].

## 5. Conclusions

In women affected by renal colic during pregnancy, the incidence of perinatal complications such as UTI, pPROM, and preterm birth was confirmed to be markedly elevated. Pregnancies complicated by renal colic should be considered as high-risk ones. For other complications such as GDM, PIH, PE, and IUGR, the results are ambiguous, and only single studies showed their increased frequency in renal colic. Further research is needed to confirm the findings. In cases of renal colic, multidisciplinary care including a urologist, gynecologist, and neonatologist is crucial for preserving fetal and maternal well-being. Moreover, it is necessary to improve the system for reporting perinatal complications and creating a multicenter database for this group of patients.

## Figures and Tables

**Table 1:** Characteristics of maternal–fetal complications in renal colic in pregnancy—A literature review.

First Author, Year of Publication	Sample Size (n)	Mean Maternal Age [years]	Mean Gestational Age [weeks]	Maternal Outcomes							Fetal Outcomes			
				PIH n (%)	PE n (%)	GDM n (%)	pPROM n (%)	UTI n (%)	Urosepsis n (%)	Cesarean Section Delivery n (%)	Premature Birth n (%)	Intrauterine Fetal Demise n (%)	NICU Admission n (%)	IUGR n (%)
Radu, V.D. et al., 2022 [1]	52	26	N/A	2 (3.9)	3 (5.8)	N/A	8 (15.4)	12 (23.1)	N/A	40 (34.8)	16 (30.8)	0	10 (19.2)	5 (9.6)
He, M.M. et al., 2022 [2]	100	31	22	N/A	N/A	N/A	N/A	22 (22.0)	N/A	38 (38.0)	8 (8.0)	N/A	N/A	N/A
Zhang, S. et al., 2016 [3]	117	25	N/A	N/A	N/A	N/A	N/A	N/A	1 (0.9)	N/A	12 (10.3)	N/A	N/A	N/A
N’gamba, M. et al., 2015 [4]	82	N/A	39	N/A	N/A	3 (3.7)	3 (3.7)	6 (7.3)	N/A	N/A	2 (2.4)	N/A	N/A	N/A
Fathelbab, T.K. et al., 2016 [5]	41	23	N/A	N/A	N/A	N/A	N/A	0	0	N/A	0	N/A	N/A	N/A
Sebastian, N. et al., 2021 [6]	11 528	<25 30.5%; 25–34 57.3%; 35+ 12.2%	N/A	430 (3.7)	536 (4.7)	778 (6.8)	73 (0.6)	N/A	N/A	4038 (35.0)	1442 (12.5)	43 (0.4)	N/A	227 (1.9)
Tangren, J.S. et al., 2018 [7]	166	32	38.7 ± 2.0	37 (22.0)	25 (16.0)	28 (18.0)	N/A	N/A	N/A	49 (30.0)	31 (18.67)	N/A	16 (10.0)	13 (8.0)

Abbreviations: PIH—pregnancy induced hypertension; PE—preeclampsia; GDM—gestational diabetes mellitus; pPROM—premature rupture of membranes; UTI—urinary tract infection; NICU—neonatal intensive care unit; IUGR—intrauterine growth restriction; N/A—not available.

## Supplementary Materials

The following supporting information can be downloaded at https://www.mdpi.com/article/10.3390/jcm13185515/s1: Table S1: Patient characteristics and the main results of the analyzed studies.
