# Supplementary material for: Maternal–Fetal Complications in Renal Colic during Pregnancy: A Scoping Review
Source: J Clin Med. 2024 Sep 18;13(18):5515. doi: 10.3390/jcm13185515 (PMC11432146; doi:10.3390/jcm13185515)
Supplement: Supplementary file 1 [file jcm-13-05515-s001.zip › jcm-3110565-supplementary.pdf]

Table S1. Patient characteristics and the main results of the analyzed studies

First author, date	Patient characteristics	Treatment description	Results	Main conclusion
Radu et al., 2022 [1]	Experimental group (N = 52)/Control group (N = 63): 26 ± 6/29 ± 6 years; N of gestations 2.4 ± 2.1/2.3 ± 1.5; parity 2.0 ± 1.9/1.9 ± 1.3; previous cesarean section N = 8/N = 10; placenta previa N = 4/N = 5; gestational hypertension N = 2/N = 6; preeclampsia N = 3/N = 1; previous renal colic N = 10/N = 2; previous nephrolithiasis N = 9/N = 1; *in vitro* fertilization N = 1/N = 1;	Experimental group: DJ ureteric stenting (insertion N = 46, replacement N = 6) – timing of the procedure (weeks of gestation / trimester of pregnancy): 23 ± 7 weeks/1st trimester N = 5, 2nd trimester N = 29, 3rd trimester N = 18; Leukocytosis pre-post: 15345 ± 2340/mm^3 → 8651 ± 1890/mm^3; CRP[1] 123.3 ± 46 mg/l; Urinalysis and urine culture: Leukocyturia N = 31, *Escherichia coli* N = 19, *Klebsiella spp*. N = 5, *Enterococcus spp*. N = 4, Serratia spp. N = 2, *Staphylococcus spp*. N = 1; Simple UHN[2] (N = 37) location: left N = 11, right N = 23, bilateral N = 3; Infected UHN (N = 13) location: left N = 1, right N = 10, bilateral N = 2; UHN grade: 1.7 ± 0.5; Urolithiasis (N = 14) location: left N = 5, right N = 8, bilateral N = 1; Urosepsis N = 15; Pyelonephritis (N = 3) location: left N = 1, right N = 2;	Treatment complications (from N = 52): Pain/urinary discomfort N = 21; Stent migration N = 3; Stent encrustation N = 2; Reflux pyelonephritis N = 2; Gross hematuria N = 1;<br><br>**Pregnancy outcome** (Experimental group / Control group): Vaginal birth N = 12/N = 35 Female newborn N = 26/N = 31; Apgar score 8.0 ± 1.6/8.7 ± 1.4; Preterm labor N = 16/N = 9; Premature rupture of membranes N = 8/N = 1; Preterm birth N = 14/N = 2; Fetal growth restriction N = 5/N = 2; Preeclampsia N = 1/N = 1; NICU[3] admission N = 10/N = 4; Post-partum UTI[4] N = 12/N = 2;	DJ stenting is a safe and effective treatment option for pregnant patients with obstructive urological disorders.

		Associated procedures: retrograde ureteroscopy with lithotripsy N = 1, endoscopic lithotripsy N = 1; Hospitalization: 5.2 ± 2.0 days; Procedure duration: 28.3 ± 13.4 min;		
Mao-Mao He et al., 2022 [2]	Early TTU* (time to ureteral stent placement < 48 h) group (N = 42)/ Delayed TTU > 48 h, (N = 58) group: Age (years) 31 ± 4.9/29 ± 4.2; BMI[5] (kg/cm^2) 23.4 ± 3.3/22.1 ± 2.9; Gestation (wks) 22 ± 7.8/22 ± 5; History of stones (%) N = 14 (53.8)/N = 12 (46.2); WBC[6] count (× 10^9/L) 14.19 ± 4.1/12.97 ± 2.9; CRP (mg/dL) 4.25/1.77; Stone size (mm) 9.98/9.07, < 10 mm, (%) N = 21 (38.2)/N = 34 (61.8), ≥ 10 mm, n (%) 21 (46.7)/24 (53.3); Hydronephrosis (mm) 47 (15, 8)/34 (15, 4), None/light, n (%) 21 (40.4)/31 (59.6), 21 (40.4), 31 (59.6); Stone location, (%): None N = 11 (45.8)/N = 13 (54.2), Ureter N = 24 (50)/N = 24 (50), Kidney N = 7 (25)/N = 21 (75); Urology consultation time (h) 19/48; ≤ 24 h (%) N = 34 (64.2)/N = 19 (35.8), > 24 h (%), N = 8 (17)/N = 39 (83);<br><br>PANP[7] group (N = 32)/No-PANP group (N = 68):	No treatment description	Early TTU (time to ureteral stent placement, < 48 h) group (N = 42)/ Delayed TTU (> 48 h, N = 58): Preoperative fetal complications, (%) N = 6 (50)/ N = 6 (50); PANP (%) N = 15 (46.9)/N = 17 (53.1); UTI after surgery (%) N = 11 (50)/N = 11 (50); LOS[8] (days) 6 ± 3/8 ± 3.6; Total cost (Yuan in RMB) 10448 ± 2412.3/13783.2 ± 6841.6; Newborn weight (g) 2891.7 ± 509.18/ 2894.5 ± 569.2; Cesarean section rate (%) N = 17 (44.7)/N = 21 (55.3); Preterm delivery (%) N= 6 (75)/N = 2 (25);<br><br>PANP group (N = 32)/ No-PANP group (N = 68): Pain to surgery (h) 114.5/84.5; < 96 h (%) N = 9 (17.6)/N = 42 (82.4); ≥ 96 h (%) N = 23 (46.9)/N = 26 (53.1); Admission to surgery (h) N = 53 (24, 160)/N = 50 (36, 90); < 48 h (%) N = 15 (35.7)/N = 27 (64.3)	For pregnant patients with renal colic, delayed surgery within 48 h is not related to the clinical outcome of the mother and child, but leads to longer hospital stay. Time from pain to hospitalization and the location of the stones are risk factors for preoperative acute pyelonephritis. The incidence of preterm birth was 8%, and 75% of preterm pregnant women suffered preoperative acute pyelonephritis.

	Age (yr) 29 ± 5/30 ± 4; BMI (kg/cm2) 21.7 ± 2.4/23.3 ± 3; Gestation (wks) 20/23; History of stones (%) 12 (46.2)/14 (53.8); WBC count (× 10^9/L) 14.6 ± 4.40/13 ± 2.8; CRP (mg/dL) 3.2/ 2.2; Stone location (%): None N = 6 (25)/N = 18 (75), Ureter N = 8 (16.7)/N = 40 (83.3), Kidney N = 18 (64.3)/N = 10 (35.7); Stone size (mm): < 10 mm (%) N = 18 (32.7)/N = 37 (67.3), ≥ 10 mm (%) N = 14 (31.1)/N = 31 (68.9); Hydronephrosis (mm): None/light (%) N = 12 (23.1)/N = 40 (76.9), Moderate/severe (%) N = 20 (41.7)/N = 28 (58.3); Urology consultation time (h): ≤ 24 h (%) N = 16 (30.2)/N = 37 (69.8), > 24 h (%) N = 16 (34)/N = 31 (66);<br><br>*early TTU (group (< 48 h, N = 42)		≥ 48 h (%) N = 17 (29.3)/N = 41 (70.7)<br>Pain to admission (h) 90/24<br>< 48 h (%) N = 3 (6.1)/N = 46 (93.6)<br>≥ 48 h (%) N = 29 (56.9)/N = 22 (43.1)<br>Preoperative fetal complications, (%) N = 6 (50)/N = 6 (50);<br>UTI (urinary tract infection) after surgery (%) N = 12 (54.5)/N = 10 (45.5);<br>LOS (days) 11 ± 5.3/7 ± 3;<br>Total cost (Yuan in RMB) 16522.6 ± 8871.61/10236 ± 3281.2;<br>Newborn weight (g) 2706.6 ± 649.5/2979 ± 460.6<br>Cesarean section rate (%) N = 14 (36.8)/N = 24 (63.2);<br>Preterm delivery (%) N = 6 (75)/N = 2 (25);	
Zhang et al. (2016) [3]	Experimental group (N = 117): Group A (persistent renal colic for 12 or fewer hours, N = 24)/ Group B (persistent renal colic for 12 to 24 hours, N = 76)/ Group C more than 24 hours, N = 17);<br>Age 25.5 ± 4.6 years; Gestation period: 9 - 36 weeks;<br>Hydronephrosis (N = 117, 100%): 1st degree (N = 66), 2nd degree (N = 39), 3rd degree (N = 12); Ureteral calculi (N = 86), obstructive	URS under general (N = 72)/ spinal (N = 45) anesthesia; 9.5F semirigid ureteroscope or guidance of a ureteral catheter (4-6F): trimester 1-2 (N = 48), trimester 3 (N = 11)/ flexible ureteroscopy: trimester (N = 16);<br>ureteroscopic DJ[10] stent insertion, no ureteral calculi (N = 31);	Threatened abortion (N = 12, rates in groups A/B/C: 8.3%/6.5%/29.4%)<br>urosepsis (N = 1, group C);<br>irritative voiding symptoms (N = 22);<br>fever (N = 5);<br>microscopic hematuria (N = 116);<br>leukocyturia (N = 37 patients);<br>positive urine culture (N = 13);<br>Uterine contractions group A/B/C: N = 2/N = 5/N = 5;	For pregnant patients with persistent renal colic/ureteral calculi and hydronephrosis, ureteroscopic DJ stent insertion and URSL are effective and safe options when conservative treatment fails, even if no urinary calculi

	ureteral calculi during ultrasonography (N = 62); mean stone diameter: 8.2 ± 0.6 mm; ureteral calculi found by URS[9] (N = 24); presence of hydronephrosis, ureteroscopy or ultrasound examination revealed no calculi (N = 31); The duration of the operation (minutes) group A/B/C: 27 ± 4.8/31 ± 4.5/22 ± 6.8;	URSL[11] with holmium laser or pneumatic ballistic (N = 86): ureteral calculi found by URS (N = 24), complete fragmentation of calculi (N = 73), stone-free rates in groups A/B/C: 87.5%/ 86.0%/77.0%; stone fragments retrograde migrated to the renal pelvis (N = 13);	Serious obstetric, fetal or urologic complications N = 0; all patients completed pregnancy to full term.	were found by ultrasound. For patients with persistent renal colic during pregnancy, the early application of ureteroscopy may reduce the risk of preterm birth.
N'gamba et al. 2015 [4]	Experimental group (N = 82); BMI 22.3 ± 0.5; Urolithiasis (N = 24); Pregnancy term: trimester 1/2/3: N = 3/N = 38/N = 41; Primipara (N = 38); median term of pregnancy (weeks): 39 (range 33 - 42); Twin pregnancy (N = 2); Hydramniosis (N = 2); Fetal macrosomia (N = 2); Medical history: Diabetes (N = 3); Renal colic (N = 27); Lithiasis (N = 9); Chronic urinary infections (N = 18); Hypothyroidism (N = 1); Pain on the left side (N = 53), right side (N = 26), bilateral (N = 3); stone locations: the pelvicalyceal system (N = 12), upper ureter (N = 2), mid-ureter (N = 2), lower ureter (N = 8); Mean right hydronephrosis 13.9 ± 1.58 mm;	Hematuria and/or leukocyturia (N = 58/38); Cytobacteriological positive urine analysis (N = 7) Abdominal ultrasonography (N = 73); Abdominal x-ray (N = 13); Abdominal CT (N = 14); Positive obstructive urolithiasis imaging (N = 24); Indications for implementing treatment: Stone size > 7 mm (N = 7); Urolithiasis complicated by urinary infections (N = 7); Unsuccessful medical pain management (N = 7); Severe hydronephrosis (N = 3); Medical treatment: Paracetamol (N = 80) NSAID[12] (N = 3);	Ureteral stenting (N = 23) Perinatal outcomes: Premature rupture of membranes (N = 3); Premature delivery (N = 2); Replacement of Double J stent before delivery (N = 5); Calcification of Double J stent (N = 6); DJ stent linked to chronic pain (N = 5); Urinary infection (N = 6); Surgical treatment required after delivery (N = 10): Extracorporeal shockwave lithotripsy (N = 6); Retrograde intrarenal surgery (N = 2); Percutaneous lithotripsy (N = 2); Stone (N = 2), calcium phosphate stone (N = 1), calcium oxalate stone (N = 1);	Identified predictive factors for a urolithiasis etiology: primiparity, leukocyturia and left hydronephrosis (> 10 mm). Obstetric consequences of acute renal colic were minor.

	Mean left hydronephrosis 5.7 ± 1.24 mm; C-reactive protein > 6mg/L (N = 26); mean renal function 149.8 ± 5;	Corticosteroids (N = 1); Phloroglucinol (N = 79); Nalbuphine (N = 49); Morphine (N = 8); Endouretheral prothesis (N = 24); Mean hospitalization time (min-max) (days) 3 (1 - 20);		
Fathelbab et al. (2016) [5]	Experimental group (N = 41); Mean age: 23 years, (range 19 – 37); Obstruction on the right side (N = 27)/left side (N = 14); Gestational age: 1st trimester (N = 4), 2nd trimester (N = 23), 3rd trimester (N = 14); Presentation: Renal colic (N = 37), hematuria (N = 23);	Ureteroscopy under epidural anesthesia (N = 41); Ureteroscopy with the presence of ureteric stones (N = 36); Mean stone size: 8.9 mm; Distal ureteric stones (N = 29);<br><br>Pneumatic lithoclast was used for stone fragmentation (N = 22), stone directly extracted (N = 4), stone migrated proximally and not accessible (N = 3), Proximal ureteric stones (N = 7), entire ureter was free of stone (N = 5);	Minor urologic complications: Mild dysuria (N = 12), Mild hematuria (N = 5);<br><br>All patients completed their pregnancy until full term without any serious obstetric complications requiring intervention.	Ureteroscopy is a safe and effective therapeutic option for the treatment of obstructing ureteral stones in pregnancy with complication rates being comparable to the non-pregnant population.
Sebastian et al. (2021) [6]	Cohort of pregnant patients (*n* = 13,792,544): Urolithiasis (*n* = 11,528)/ No urolithiasis (*n* = 13,781,016); Age (years), n (%) < 25: 30.47/33.74,; 25 – 34: 57.29/51.72; >35: 12.24/14.54; Race, n (%) Caucasian 75.01/52.99; Black 4.84/13.70; Hispanic 14.20/22.84; Other 5.95/10.47; Risk factors, n (%): Preexisting diabetes 0.82/0.88,	No treatment description	Maternal outcomes, urolithiasis/no urolithiasis (%); antepartum: Gestational hypertension 3.73/3.20; Preeclampsia/Eclampsia 4.65/3.55; Gestational diabetes mellitus 6.75/5.27; pPROM 0.63/0.60; placental abruption 1.51/1.07; placenta previa 0.82/0.53; Intrapartum: chorioamnionitis 1.78/1.83; Cesarean delivery 35.03/30.23; Instrumental	Although the mechanism is unclear, women with urolithiasis in pregnancy are at an increased risk of adverse pregnancy and neonatal outcomes.

	Hypertension 0.82/1.59, Smoker 9.00/5.15, Obesity 3.05/1.81, Morbid obesity 1.20/1.02;		delivery 4.95/6.07; Postpartum: Postpartum hemorrhage 2.96/2.76; Pyelonephritis 5.460.07/88.87, Venous thromboembolism 0.38/0.20, Maternal death 0.03/0.01; Neonatal Outcomes: Congenital anomalies 1.43/0.43; IUGR 1.97/2.01; Preterm birth 12.51/7.09;	
Tangren et al, 2018 [7]	Analyzed group (N = 1430): stone formers (N = 166)/ non-stone formers (N = 1264); Age at first prenatal visit, yr 32 ± 65/32 ± 66; Mean gestational age at delivery (wks): 38.7 ± 2.0/39.2 ± 1.7; Nonwhite race, n (%): 84 (51)/597 (47); Body mass index, (kg/m2): 27.66/27.66; Nulliparous, n (%): 49 (30)/393 (31); Parity: 1 [0 – 2]/1 [0 – 2]; Imaging modality, n (%) Ultrasound 50 (30)/ 386 (29); Computed tomography scan: 116 (70)/896 (71); Imaging location, n (%): Inpatient 22 (13)/64 (13); Ambulatory 104 (62)/702 (56); Emergency room 40 (24)/398 (31); Multiple or bilateral stones, n (%) 73 (44)/not applicable; Hydronephrosis, n (%) 35 (21)/not applicable; Time from imaging to conception, (yr) 1.9 [0.8 – 4.2]/2.2 [0.9 – 4.3]; Preconception laboratory values, (mg/dl) Serum creatinine: 0.7360.16 (n =	No treatment description	Main pregnancy outcomes (stone formers/ non-stone formers): Metabolic complications of pregnancy: Average glucose, (mg/dl): 111 ± 18 /107 ± 18 0.04; Gestational diabetes, n (%): 28 (18)/ 65 (6); Gestational weight gain, (kg): 11 ±6.0/12 ± 5.2 0.03; Hypertensive complications of pregnancy: Maximum systolic BP (mm Hg) 126 ±14/121 ± 10; Maximum diastolic BP (mm Hg) 78 ± 9/77 ± 8; Gestational hypertension, n (%): 37 (22)/162 (13); Preeclampsia, n (%): 25 (16)/70 (8); Cesarean section, n (%): All 49 (30)/ 408 (32), Elective 13 (27)/192 (48); Main neonatal outcomes: Preterm delivery (week) n (%) < 37: 27 (16)/96 (8); < 34: 4 (3)/16 (1);	In women without preexisting diabetes, hypertension, and chronic kidney diseases, a history of nephrolithiasis was associated with gestational diabetes and hypertensive disorders of pregnancy, especially in women with high first-trimester body mass index.

	166)/0.7260.15 (n = 1264); Serum calcium 9.260.5 (n = 91)/9.360.4 (n = 711); Uric acid 4.361.2 (n = 83)/4.561.5 (n = 372);		Baby weight, (g): 3286 ± 634/3415 ± 541;<br>Small for gestational age, n (%): 13 (8)/86 (7);<br>Large for gestational age, n (%): 15 (9)/122 (10);<br>Neonatal ICU admission, n (%):16 (10)/77 (6);<br>Composite fetal outcome, n (%): 35 (21)/191 (15);	

Legend:
[1] CRP – C-reactive protein
[2] UHN – ureterohydronephrosis
[3] NICU – Neonatal Intensive Care Unit
[4] UTI – urinary tract infection
[5] BMI – Body Mass Index
[6] WBC – White blood cells
[7] PANP – preoperative acute pyelonephritis
[8] LOS – length of hospital stay
[9] URS – ureteroscopy
[10] DJ – Double J
[11] URSL – ureteroscopic lithotripsy
[12] NSAID – Non-steroidal anti-inflammatory drugs
